# Assessment of sulfonated homo and co-polyimides incorporated polysulfone ultrafiltration blend membranes for effective removal of heavy metals and proteins

**DOI:** 10.1038/s41598-020-63736-8

**Published:** 2020-04-27

**Authors:** Mohammad A. Jafar Mazumder, Panchami H. Raja, Arun M. Isloor, Muhammad Usman, Shakhawat H. Chowdhury, Shaikh A. Ali, Inamuddin, Amir Al-Ahmed

**Affiliations:** 1 Chemistry Department, King Fahd University of Petroleum & Minerals, Dhahran, 31261 Saudi Arabia; 2 Membrane Technology Laboratory, Chemistry Department, National Institute of Technology Karnataka, Surathkal, Mangalore, 575 025 India; 3 Center for Research Excellence in Nanotechnology (CENT), King Fahd University of Petroleum & Minerals, Dhahran, 31261 Saudi Arabia; 4 Department of Civil and Environmental Engineering, King Fahd University of Petroleum & Minerals, Dhahran, 31261 Saudi Arabia; 5 Chemistry Department, Faculty of Science, King Abdulaziz University, Jeddah 21589, Saudi Arabia; 6 Advanced Functional Materials Laboratory, Department of Applied Chemistry, Faculty of Engineering and Technology, Aligarh Muslim University, Aligarh, 202 002 India; 7 Center of Research Excellence in Renewable Energy, King Fahd University of Petroleum & Minerals, Dhahran, 31261 Saudi Arabia

**Keywords:** Pollution remediation, Pollution remediation

## Abstract

Sulfonated homo and co- polyimide (sPI) were synthesized with new compositional ratios, and used as additives (0.5 wt%, 0.75 wt%, and 1.0 wt%) to prepare blend membranes with polysulfone (PSf). Flat sheet membranes for ultrafiltration (UF) were casted using the phase inversion technique. Surface morphology of the prepared UF membranes were characterized by atomic force microscopy (AFM) and scanning electron microscopy (SEM). Surface charge of the membranes were determined by zeta potential, and hydrophilicity was studied by contact angle measurement. The contact angle of the membrane decreased with increasing sPI additive indicates increasing the hydrophilicity of the blend membranes. Filtration studies were conducted for rejection of heavy metals (Pb^2+^ and Cd^2+^) and proteins (pepsin and BSA). Blend membranes showed better rejection than pure PSf membrane. Among the blend membranes it was observed that with increasing amount of sPIs enhance the membrane properties and finally, PSf-sPI5 membrane with 1 wt% of sPI5 showed the improved permeability (72.1 L m^−2^ h^−1^ bar^−1^), and the best rejection properties were found for both metal ions (≈98% of Pb^2+^; ≈92% of Cd^2+^) and proteins (>98% of BSA; > 86% of Pepsin). Over all, this membrane was having better hydrophilicity, porosity and higher number of sites to attach the metal ions. Its performance was even better than several-reported sulfonic acid based UF membranes. All these intriguing properties directed this new UF membrane for its potential application in wastewater treatment.

## Introduction

Worldwide water pollution is a major concern and wastewater treatment has become one of the top priority for both industries and public sectors. Among the different pollutants that are going into the water bodies, toxic heavy metals had received extreme urgency because of their acute toxicities and carcinogenic nature^1,2^. Number of methods are available to remove the heavy metals, such as, chemical precipitation, ion exchange, reverse osmosis (RO), electro-dialysis, adsorption, nanofiltration, coagulation, flocculation, floatation, etc.^3,4^. However, these methods have several disadvantages such as, high reagent requirement, unpredictable metal ion removal, generation of toxic sludge, etc.^2,3,5^. Among the different membrane based wastewater treatment techniques, ultrafiltration (UF) and RO process have received considerable attention, as they are capable of removing not only suspended solid and organic compounds but also inorganic contaminants such as heavy metals. UF method is much cheaper and at the same time, it is a low energy process. Polymeric membrane is the core of the UF separation technology. Since this process is effective and already gains much interest in academic and industrial research, in the this article, the primary effort has been made to develop new blend membrane with better properties to remove non-biodegradable and toxic heavy metal ions^3,6^ and at the same time protein based organic pollutants^7–9^.

Blending of polymers is an easy and effective approach to incorporate novelty into the materials with a broad diversity of properties between those of pure components. This process not only modifies the properties of the membrane but also increases the flux of the membrane^10,11^. In addition, researchers have also use this opportunity to further improve the surface wettability, hydrophilicity, and surface charge of the membrane by incorporating different functional groups, such as, hydroxyl (–OH), amine (–NH_2_), sulfonic (–SO_3_H), and carboxylic acids (–COOH) into the backbone of the polymer^11,12^. Though very few sulfonic acid group modified membranes are used in the UF technology, but in the literature, researchers pointed out that the introduction of sulfonic acid group in a polymer is one of the effective approach to increase hydrophilicity and other membrane properties, such as, higher water flux, improved permeability, etc.^10,13^. Moreover, during the UF membrane fabrication process, polymeric additives such as polyvinylpyrrolidone (PVP) and polyethylene glycol (PEG) are commonly used to control the pore structure^2,6,10^. This naturally helps to enhance the viscosity, improve hydrophilicity, enhance the pore formation, and suppress macro-void formation in order to create membranes with better rejection and higher pure water flux.

There are several reports where sulfonated polymers were blended with other polymers to prepare UF membranes with improve properties^14–16^. Jacob *et al*.^10^, dissolved sulfonated polyethersulfone (sPES)/polysulfone (PSf) in *N*-methyl-2-pyrrolidone (NMP) and prepared flat sheet membranes by dry–wet phase inversion technique. They used PEG-200 as a non-solvent additive in the casting solution to control the porosity, and studied the effect of sPES/PSf blending weight ratio on the morphology, hydrophilicity, water content, porosity, hydraulic resistance, pure water flux, compaction, and molecular weight cut-off (MWCO) of the prepared membranes. Attractive asymmetric microstructure was observed with a thin skin layer and porous sub-layer with significant improvement of membrane performance. When the membranes were subjected to heavy metals rejection study for the Cu^2+^ and Zn^2+^ in a polymer enhanced UF (PEUF) technique, in presence of polyethyleneimine (PEI) as complexing agent, the blend membrane showed better rejection capacity then the membrane with pure PSf. They also observed that the Cu^2+^ rejection rate was much higher than that of the Zn^2+^. This was attributed to the better binding capacity of the Cu^2+^ ion with PEI, and which forms stronger complexes with stable bonds^17^, whereas the Zn^2+^ complexes have low ligand field stability^18^. In another work, Loredo *et al*.^11^, prepared sulfonated poly(etherimide) (sPEI) by treating poly(etherimide) with acetyl sulfate (a sulfonating agent), and used this sPEI membrane for dialysis process. They found that this sPEI membrane had better thermal stability even that of Nafion®. They also observed that the sulfonic groups present in the polymeric matrix facilitates the cation transportation. In another work, Santiago *et al*.^12^, synthesized series of new sulfonated fluorine-containing aromatic polyamides with increasing degree of sulfonation (DS). They prepared membranes using these sulfonated polyamides and studied heavy metals (Pb^2+^ and Hg^2+^) removal capacity in an adsorption based removal method. Adsorption capacities of all these membranes toward Pb^2+^ ions were found to be higher than that of the Hg^2+^ ions, and this tendency increases with increasing DS. The equilibrium adsorption amounts were determined to be 11.87 mg g^−1^ for Pb^2+^ and 5.17 mg g^−1^ for Hg^2+^ ions for the membrane with highest DS. Several other researchers also prepared sulfonated polymer based membranes^14,19^, and studied their properties for UF process.

In this work, sulfonated polyimide (sPI) polymers were synthesized from sulfonic acid containing monomers in the homo- (sPI4) and co- (sPI5) polymerization process in a new compositional ratio. These sPIs were used as additives to prepare the blend membranes with polysulfone. PSf was selected as the base polymer due to its good mechanical strength and film-forming properties. In addition, these polymers are stable over a wide pH range, which is considered as an important parameter of the membrane for UF process. Here, PVP was used as a non-solvent additive to control the pore formation. The effects of compositional ratios of the blended polymers on the surface morphology, water content, hydraulic resistance, hydrophilicity and pure water flux were studied, and compared with membrane prepared from pure PSf. Furthermore, the performance of these membranes on the rejection and permeate flux of toxic heavy metal ions, such as, Pb^2+^ and Cd^2+^ and proteins, such as pepsin and BSA were investigated.

## Experimental

### Materials

1,4,5,8-naphthalenetetracarboxylic dianhydride (NTDA), and 4,4′-diaminodiphenyl ether (ODA) were purchased from Sigma Aldrich, and were used as received. Concentrated sulfuric acid (95%), fuming sulfuric acid (SO_3_, 60%), *m*-cresol and polyethyleneimine (Mn ~60 kDa) were purchased from Fisher Scientific, and used as received. Triethylamine (Et_3_N) obtained from BDH Chemical Ltd. (Pool, England), distilled and dried with 4A molecular sieve prior to use. Silica gel 100 was purchased from Fluka Chemie AG. Polysulfone (PSf, Udel P-3500), polyvinylpyrrolidone (PVP-K30), and *N*-methyl-2-pyrrolidone (NMP) (purity > 99.5%) were obtained from Acros Organics. The PVP and NMP were dried at 100 °C for 24 h before use. Analytical grade lead nitrate, cadmium nitrate tetrahydrate, pepsin and bovine serum albumin (BSA) were procured from Sigma Aldrich. All solvents were of reagent grade (Sigma Aldrich). Water was distilled and then de-ionized using a Milli-Q system from Millipore.

### Physical Methods

Digital melting point apparatus (Electro thermal- IA9100) recorded the melting points using heating rates of 1 °C min^−1^ near the melting points. The structural composition of the synthesized compounds was determined by Perkin Elmer 16 F PC Fourier transform infrared (FT-IR) spectrometer (spectral resolution: 4 cm^−1^, number of scans: 16), and ^1^H and ^13^C NMR using DMSO-*d*_6_ on a Bruker 400 MHz spectrometer. The elemental composition was determined on a Perkin Elmer Elemental Analyzer (Series 11 Model 2400) (Waltham, Massachusetts, USA). A TA Instruments SDT Q600 thermogravimetric analyzer was used to perform the thermogravimetric analysis (TGA) under nitrogen (N_2_; flow rate of 50 mL min^−1^) using a matched platinum/platinum–rhodium thermocouple pair, and increasing the temperature from 20−800 °C by 10 °C min^−1^. Size exclusion chromatography (SEC) was performed using a MCX column connected with a Viscotek SEC system, and was calibrated against narrow molecular weight polyethylene glycol standards. The polymer solution was prepared in 0.05 M of LiCl and 0.05 M of phosphoric acid, and NMP was used as an eluent.

### Synthesis of additives

#### Synthesis of 4,4′-Diaminodiphenyl Ether-2, 2′-disulfonic Acid (DADPEDS)

The DADPEDS was synthesized following a modified literature procedure reported elsewhere^13^. Briefly, 5.00 g (25.0 mmol) of 4,4′ diaminodiphenyl ether (ODA) was transferred into a 50 mL round bottom (RB) flask in ice bath. 8.0 mL (77.5 mmol) of concentrated sulfuric acid (95%) was slowly added to the RB flask while stirring, and dissolve the ODA. 18 mL (135 mmol) of fuming (SO_3,_ 60%) sulfuric acid was slowly added with stirring the reaction mixture at 0 °C. The reaction was continued for 2 h at 0 °C, then raised to 80 °C, and continue the reaction for 4 h. Upon completion of the reaction, the reaction mixture was cool to room temperature, and then transferred into 30 g of crushed ice for the formation of precipitates. The resulting precipitate was filtered and dissolved in 20 mL 1 M NaOH. The basic solution was filtered and filtrate was acidified by 5 mL of concentrated HCl (37%), solid precipitate formed. The solid precipitates were filtered and sequentially washed with 5 mL of water and 5 mL of methanol, and then dried to a constant weight under vacuum (∼30 mm Hg) at 70 °C. Yield: (15.6 g, 87%). Mp 266.3 °C. Elemental analysis of C_12_H_12_N_2_O_7_S_2_ found C, 40.6; H, 3.5; N, 7.6; S, 17.4; requires C, 40.0; H, 3.4; N, 7.8; S, 17.8.

#### Synthesis of NTDA- DADPEDS homopolyimides (sPI4)

The sPI4 homopolyimides was synthesized following a modified published literature procedure^13^. To a 100 mL RB flask, 5.41 g (15.0 mmol) of DADPEDS and 50 mL of *m*-cresol was added with stirring. 3.6 g (36.1 mmol) of Et_3_N was slowly added to the RB flask under N_2_, and dissolve the DADPEDS. 4.03 g (15.0 mmol) of NTDA and 2.60 g (21.4 mmol) of benzoic acid were added under N_2_, the reaction mixture was then stirred at 25 °C for 30 min, followed by heated at 80 °C for 4 h and 180 °C for 18 h. After completion of the reaction, the crude reaction mixture was cool to 75 °C, and 90 mL of *m*-cresol was added to dilute the reaction mixture. The reaction mixture was then slowly poured into the 400 mL of acetone, which results fiber-like precipitates. The fiber-like precipitates were filtered and wash with an additional 500 mL of acetone, and then dried to a constant weight under vacuum (∼30 mm Hg) at 70 °C. Yield: (11.6 g, 92%).

#### Synthesis of ODADS Based Copolyimides (sPI5)

The sPI5 copolyimides were synthesized following a procedure described as above (synthesis of sPI4) with slight modifications. Briefly, to a stirring solution of 3.61 g (10.1 mmol) of DADPEDS and 60 mL of *m*-cresol, 2.40 g (24.0 mmol) of Et_3_N was slowly added to the 100 mL RB flask under N_2_. After DADPEDS was completely dissolved, 2.01 g (10.0 mmol) of non-sulfonated ODA, 5.36 g (20.0 mmol) of NTDA and 3.41 g (28.0 mmol) of benzoic acid were added. The resultant mixture was stirred at 25 °C for 30 min, and then heated at 80 °C for 4 h and 180 °C for 18 h. After the time elapsed, the crude reaction mixture was cool to 75 °C; an additional 120 mL of *m*-cresol was added to dilute the highly viscous solution, which was then slowly poured into 800 mL of acetone. The fiber-like precipitate was filtered off, washed with acetone (1000 mL), and dried to a constant weight under vacuum (∼30 mm Hg) at 70 °C. Yield: (10.3 g, 89%).

### Degree of Sulfonation (DS)

The DS was determined by titration method^20^. Briefly, 200 mg of dry sulfonated polymer (sPI4 or sPI5) was dissolve in 10 mL of DMSO. The sulfonated polymer containing solution was then titrated against 0.1242 M NaOH using phenolphthalein as an indicator. The DS of the additives was calculated using the Eq. (1):

1 $$DS( \% )=\frac{\frac{Mw}{1000}\times {C}_{m}\times {\rm{V}}\times 100}{{\rm{Wp}}-\frac{Ms}{1000}\times {C}_{m}\times {\rm{V}}}$$

where, M_w_ is the molecular weight of sPI4 or sPI5 repeat unit, M_s_ is the molecular weight of –SO_3_H group, C_m_ is the molar concentration of standard NaOH solution (mol L^−1^); V is the volume of NaOH used to neutralize the polymer solution in mL, and W_p_ is the weight of the polymer sample in g.

### Preparation of blend flat sheet membranes

Calculated amount of polymer additive sPI4 was dissolved in DMSO. The dried PSf and PVP were dissolved in NMP. These two solutions were mixed slowly under stirring for 24 h at 60 °C to obtain a homogeneous solution. The resultant solution was degassed in an ultrasonic bath to remove trapped air bubbles. Finally, the flat sheet blended membranes (PSf-sPI4) were prepared by phase inversion method^21^. The obtained membranes were immersed in 10% glycerol for 24 h followed by an immersion in de-ionized water for 6 h to remove all trapped solvent molecules from the membrane. Similar procedure was used to prepare the blend membrane (PSf-sPI5) with sPI5 additive. The weight percent compositions of different constituents are presented in Table 1. Pure PSf based membranes were also prepared for comparative study.

**Table 1:** The composition of membrane casting solution.

Membranes	PSf (wt%)	NMP (wt%)	PVP (wt%)	sPI4 or sPI5 (wt%)	DMSO (wt%)
Pure PSf	17.00	50	2	0	31
sPI4 or sPI5 (0.5 wt%)	16.50	50	2	0.50	31
sPI4 or sPI5 (0.75 wt%)	16.25	50	2	0.75	31
sPI4 or sPI5 (1 wt%)	16.00	50	2	1.00	31

### Membrane Characterization

#### Surface morphologies and surface roughness

Impressions of surface roughness of the prepared membranes were analyzed using Bruker atomic force microscope (AFM) (Innova SPM). Membrane samples were cut into small pieces (Area 0.25 cm^2^), and were attached to a glass plate of area 0.50 cm^2^ with the help of two-sided tape. The prepared membranes were scanned by the AFM tapping tool with a size range of 3 × 3 μm^2^. The cross-section morphology of the fabricated flat sheet blend membranes was studied by a scanning electron microscopy (SEM) (JEOL JSM-6380LA). The dried membranes were dipped in methanol solution for 3 min to avoid surface charging of the membranes, and the membranes were fractured using liquid N_2_^22^. Finally, all the membranes were coated with Pt using an EMITECH K575 sputter coater before imaging.

#### Hydrophilicity

The surface hydrophilicity of all the membranes was investigated using FTA-200 dynamic contact angle measurement in a sessile droplet method^7^. De-ionized water was used as probe liquid. The source of light was focused on one side of the instrument on hand camera, which was used to capture an image of the bubble on the surface of the membrane. The contact angle was measured at three different places and the average value was reported.

#### Water uptake and porosity

Investigation of water uptake study was performed on prepared membranes following the literature procedure^9^. The membrane samples were cut into small pieces with a diameter of 2 cm^2^. The dried membranes were immersed in de-ionized water for 24 h. The membranes were then taken out from de-ionized water, and wet weight (W_w_) was noted after wiping with a blotting paper. After measuring the wet weight, the membranes were allowed to dry in an oven at 60 °C for 6 h, and dry weight (W_d_) of the membranes was noted. Percentage of water uptake for the individual membranes was calculated using Eq. (2)

2 $${\rm{Water}}\,{\rm{Uptake}}\,( \% )=\frac{({W}_{w}-{W}_{d})\times 100}{{W}_{w}}$$

The porosity of the membranes was calculated using Eq. (3)

3 $${\rm{P}}( \% )=\frac{({W}_{w}-{W}_{d})\times 100}{({\rho }_{w}\times A\times \delta )}$$

where, ‘A’ is an area of the wet state of the membrane in m^2^, ‘δ’ is membrane thickness in m, and ‘ρ_w_’ is pure water density (0.998 g cm^−3^).

#### Pure water flux and Antifouling stduy

The pure water flux (PWF) of the membranes was measured using a self-constructed lab scale dead end filtration cell. The membranes (area = 5 cm^2^) subjected to a pure water permeation experiment, where pure water was used as the feed. The permeate sample collection was started after 15 min of exposure to a 0.5 MPa *trans*membrane pressure (TMP), and continued at every 15 minutes interval with 0.4 MPa TMP. The PWF (*J*_w_) was calculated using Eq. (4):

4 $${J}_{w}=\frac{Q}{\Delta {\rm{tA}}}$$

where *J*_w_ is expressed in L m^−2^ h^−1^ and *Q* is the amount of water collected during a Δ*t* (h) time interval using a membrane of area *A* (m^2^).

Antifouling performances of prepared membranes were performed as described elsewhere in the literature^22^. In brief, initially, PWF study was conducted, and then BSA protein solution (800 mg L^−1^) was used to examine the membrane antifouling experiments. BSA solution was reserved in feed tank and BSA flux operated at 0.4 MPa TMP with 15 min interval for 120 min. The BSA permeability ‘J_p_’ (L m ^−2^ h^−1^) values for each membrane was noted. The membranes were then cleaned with distilled water and again PWF were performed under same conditions as mentioned above, and ‘J_w2_’ (L m ^−2^ h^−1^) values of the pure water permeability were noted. The fouling feature of membranes, flux recovery ratio (FRR) was measured by Eq. (5),

5 $${\rm{FRR}}( \% )=\frac{{{\rm{J}}}_{{\rm{w}}2}}{{{\rm{J}}}_{{\rm{w}}1}}\times 100$$

The fouling impact on the membranes was further analyzed by reversible fouling ratio (Rr) and irreversible fouling ratio (R_ir_) by the following Eqs. (6, 7);

6 $${{\rm{R}}}_{{\rm{r}}}( \% )=\frac{{{\rm{J}}}_{{\rm{w}}2}-{{\rm{J}}}_{{\rm{p}}}}{{{\rm{J}}}_{{\rm{w}}1}}\times 100$$

7 $${{\rm{R}}}_{{\rm{ir}}}( \% )=\frac{{{\rm{J}}}_{{\rm{w}}1}-{{\rm{J}}}_{{\rm{w}}2}}{{{\rm{J}}}_{{\rm{w}}1}}\times 100$$

#### Determination of surface charge of the membrane

Zeta potential of the selected blend membranes (PSf-sPI4 (1 wt%) and PSf-sPI5 (1 wt%)) were analyzed in the electrokinetic analyzer (Surpass Anton Paar) by streaming current method^23^. The flat sheet membranes were cut into the area of 2 cm × 1 cm, and placed on the adjustable gap cell. Successively, 0.001 M KCl was used as the background electrolyte and circulated on the measuring cell. Manual titrations method with 0.1 M HCl and 0.1 M NaOH were used to contemplate the pH-dependent analysis of zeta potential. The zeta potential graph of the two blend membranes were plotted to analyze the surface charge.

### Rejection performance of membranes

#### *Pb*^*2+*^*and Cd*^*2+*^*removal study*

Heavy metal ion rejection performance of the blend membranes were studied by polymer enhanced UF (PEUF) method. For the PEUF process, aqueous solutions of Pb^2+^ and Cd^2+^ were prepared at an initial concentration of 500 ppm with 1 wt% of the PEI, and pH of the solutions was adjusted to 6.25 by standardized 0.1 M HCl or 0.1 M NaOH^24^. Solutions containing metal ions and PEI were mixed thoroughly and left standing for 3 days for completion of binding between metal ions and PEI. PEI complexed metal ion solutions were filtered through the membranes and the permeate was collected. The percent rejection of the metal ions by the membranes during the filtration was determined by analyzing the concentration of feed and permeate solution using an Atomic Absorption Spectrophotometer (GBC 932 Plus). The percentage of metal ions rejected by the membrane was calculated using Eq. (8),

8 $${PercentRejection}( \% {R})=\left(1-\frac{{C}_{p}}{{C}_{f}}\right)\times 100$$

where, C_*p*_ (mg mL^−1^) and C_*f*_ (mg mL^−1^) are the concentrations of permeate and feed solutions, respectively.

#### Protein rejection

Protein rejection study was performed in a cross-flow filtration unit incorporated with the polymeric membranes. In present study, 1000 ppm concentration of pepsin and BSA protein solutions were prepared, and the pH of the solutions were adjusted to 6.8 ± 0.4^25^. The rejection ability of all the membranes were determined at 25 °C and 0.4 MPa TMP using a 45 minutes time duration. Further, the feed and permeate sample were assessed by UV–Vis spectrophotometer (HACH, DR/5000 instrument). The protein samples permeates were calibrated at different conditions. The maximum absorbance was recorded at a wavelength of 250 nm for pepsin, and 278 nm for BSA. The percentage of protein rejected by the membrane was calculated by Eq. (8).

## Results and discussion

### Synthesis and characterization of sulfonated polyimides

**Figure 1:**
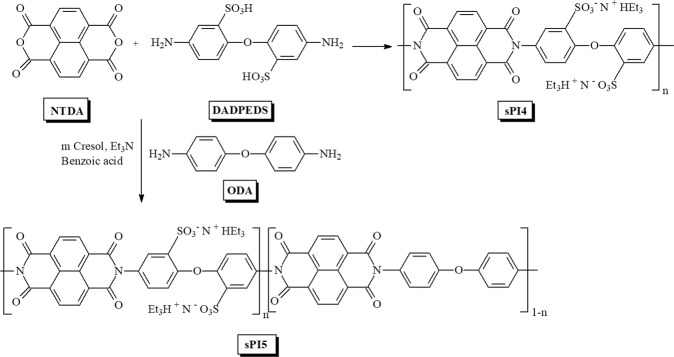
Synthesis of sPI4 and sPI5.

Modified monomer DADPEDS (2) was prepared by the reaction between 4,4′ diaminodiphenyl ether ODA (1) and fuming sulfuric acid with good yields. The homo-polyimides (sPI4) were synthesized by reacting DADPEDS (2) with NTDA (3) in presence of Et_3_N and benzoic acid as catalysts (Fig. 1). Similarly, the co-polyimides (sPI5) were synthesized by reacting (ODA) (1), DADPEDS (2) and NTDA (3) with an excellent yield (Fig. 1).

**Figure 2:**
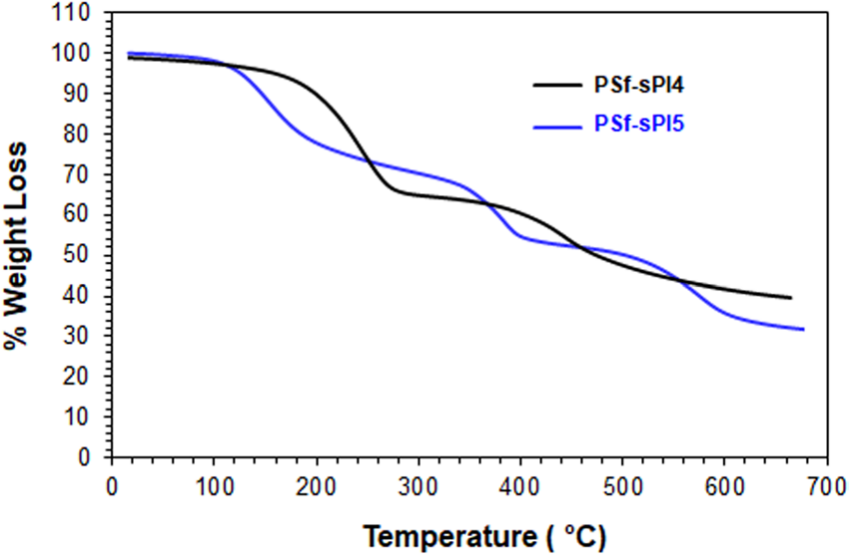
TGA curve of polyimides sPI4 and sPI5.

NMR and FTIR successfully characterized the chemical structure of the monomer and sPI’s. The detail structural compositions are depicted in supporting information (Figs. S1–S3). The molecular weight of the homo- (sPI4) and co- polyimides (sPI5) were determined by GPC. The number and weight average molecular weight (M_n_, M_w_) of these polyimides were found to be (23, 58) kDa and (25, 68) kDa, respectively. The DS was determined by titrimetric method, and the values were calculated to be 49.5% and 67.6% for sPI4 and sPI5, respectively. The thermal stability of the sPI4 and sPI5 (dried under vacuum at 60 °C for 12 h) were determined by TGA, and are depicted in Fig. 2. The TGA curve shown in Fig. 2 clearly revealed good thermal stability and no sudden weight loss was observed up to 200 °C.

### Morphology of membrane surface and cross-sections

**Figure 3:**
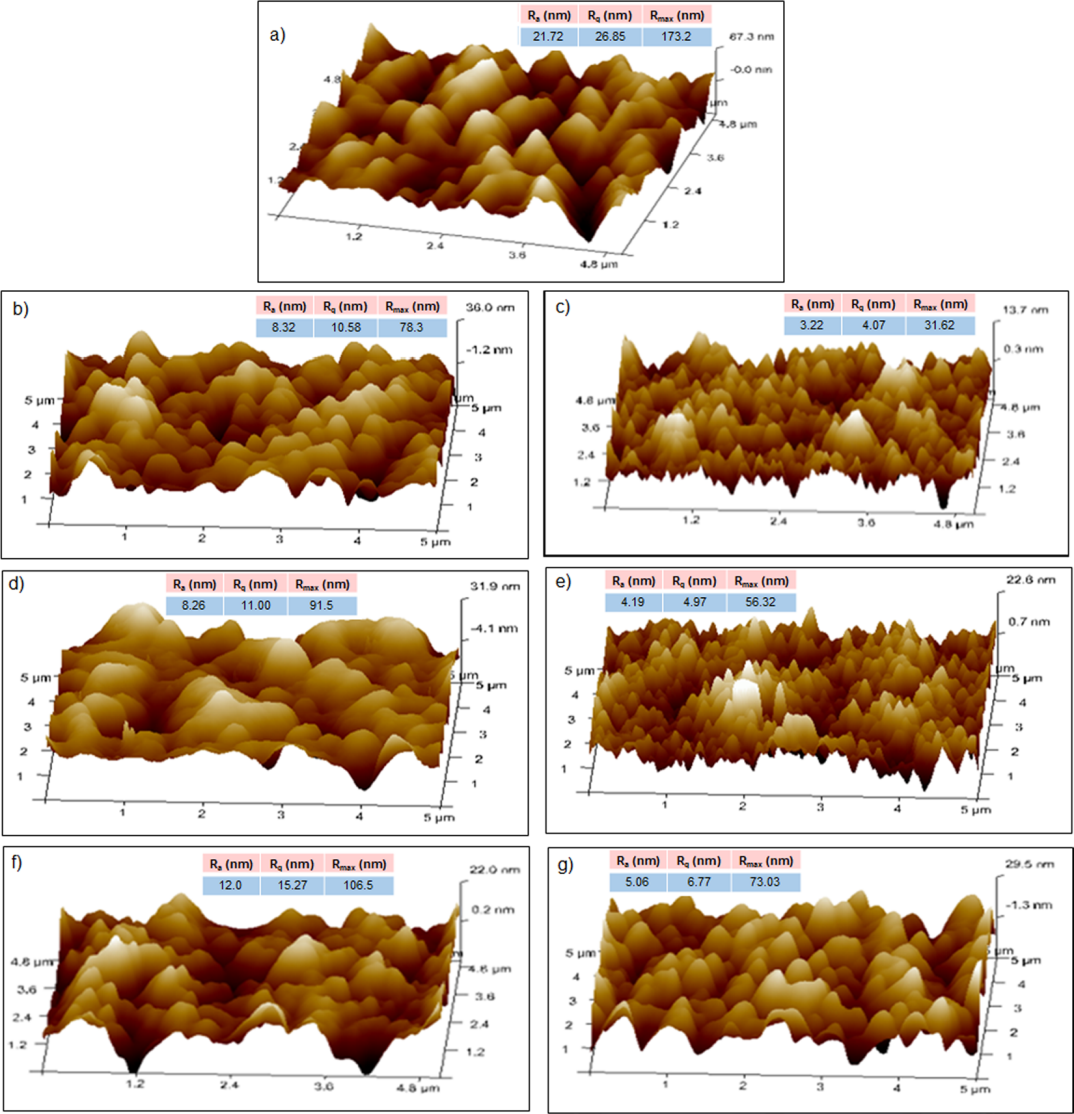
The 3D AFM images of the (**a**) neat membrane, and (**b,c**) 0.5 wt%, (**d,e**) 0.75 wt% and (**f,g**) 1 wt% PSf-sPI4 and PSf-sPI5 blend membranes, respectively. The surface roughness parameters of membranes are inserted inside the figures.

3D AFM topological images of the PSf and blend membranes are presented in Fig. 3. The surface roughness (R_a_), root mean square Z- data (R_q_) and the height difference between five maximum height peaks and five minimum height peaks (R_z_) are embedded inside Fig. 3. It is clear from the images that the surface of the pure PSf membrane is found to be very rough as compare to the blend membranes. However, with the increasing amount of additives, the surface roughness decreases, which could be due to the non-homogeneous dispersion at higher concentration. The PSf-sPI5 samples have shown smoother surface as compare to the PSf-sPI4 blend membranes (Fig. 3, inserted table), which attributed to the higher DS value and better homogeneity during blending^26,27^.

**Figure 4:**
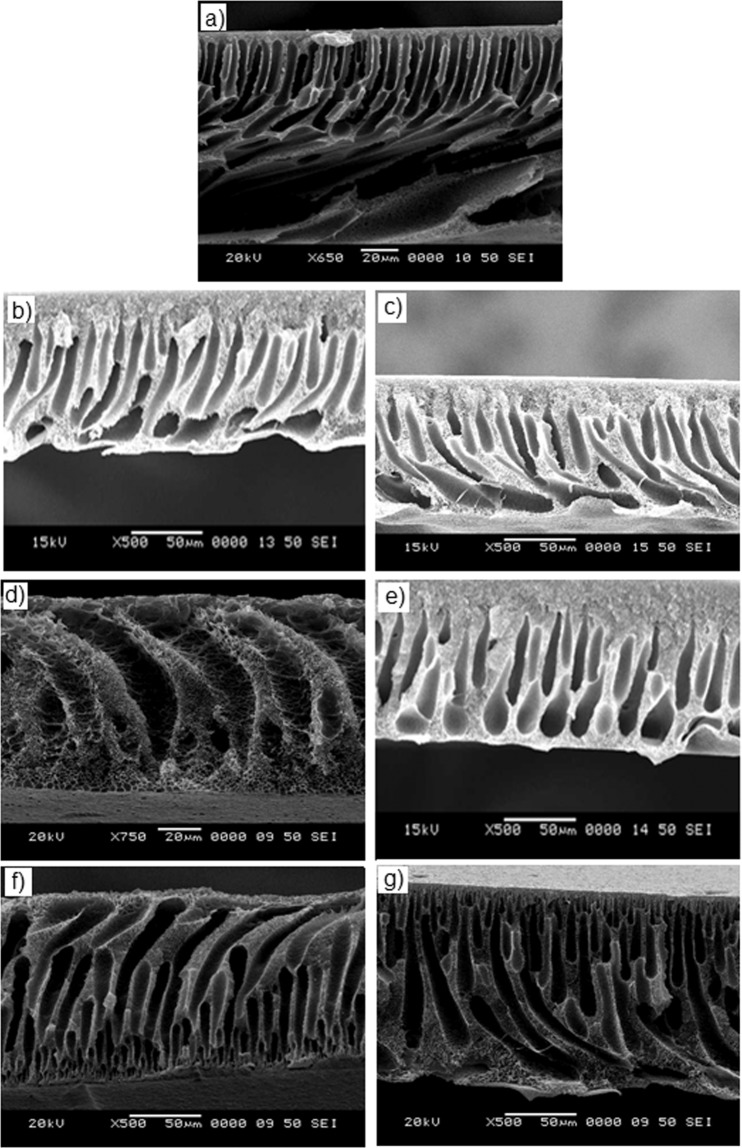
Cross-section SEM images of (**a**) neat membrane, and (**b,c**) 0.5 wt%, (**d,e**) 0.75 wt% and (**f,g**) 1 wt% PSf-sPI4 and PSf-sPI5 blend membranes, respectively.

Cross-section SEM images of pure PSf and blend membranes are depicted in Fig. 4. The fabricated membranes had an asymmetric structure with a dense skin layer, followed by a layer of fingerlike pores that further merge into macro-voids at the bottom, similar to other reported works on blend membranes with sulfonated polymers as additives^10,14^. Increasing hydrophilicity of the membrane normally enhance the phase separation process by increasing the affinity of the polymer for the non-solvent (water) during the coagulation process. This affinity helps the formation of the finger-like structures in the membrane sub-layer. From Fig. 4f,g, it was found that with increasing the amount of additives (sPI4 or sPI5) into the blends, hydrophilicity increases. Moreover, the number of finger-like structures appears in higher numbers and the fingers become thinner and longer, which is beneficial for the quicker pass of water molecules^19,28^. Therefore, it can be articulated that the separation layer was getting thicker and denser with the increasing amount and/or DS of the polymer. That is why with 1 wt% additives, an extreme pore structures was observed (Fig. 4f,g) as compared to that of pure PSf membranes (Fig. 4a)^14,29,30^.

### Membranes hydrophilicity, water uptake, porosity and permeability study

The effects of sulfonated additives on membranes water uptake, porosity and permeability values are summarized in the Table 2.

**Table 2:** Membranes properties.

Membranes	Water uptake (%)	Porosity (%)	Permeability (L h^−1^m^-2^ bar^−1^)
Pure PSf	35.2	25.5	20.2 ± 2.1
PSf-sPI4 (0.5 wt%)	46.5	26.8	26.2 ± 1.5
PSf-sPI4 (0.75 wt%)	44.6	28.0	30.1 ± 0.8
PSf-sPI4 (1 wt%)	54.3	33.2	41.0 ± 1.0
PSf-sPI5 (0.5 wt%)	48.2	23.6	25.5 ± 0.9
PSf-sPI5 (0.75 wt%)	55.8	29.0	33.2 ± 1.1
PSf-sPI5 (1 wt%)	71.3	38.6	72.1 ± 0.4

#### Hydrophilicity

**Figure 5:**
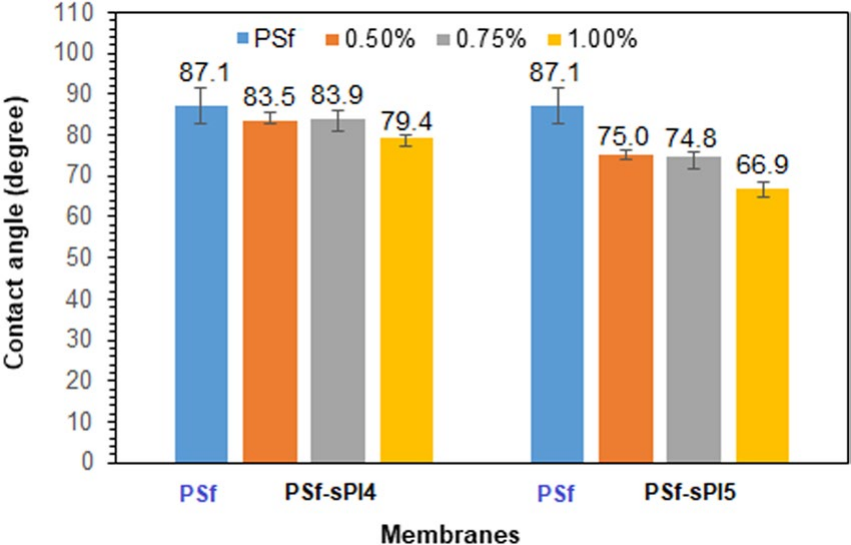
Water contact angle comparison bar chat of the neat and PSf-sPI blend membranes.

Water contact angle of the PSf and blend membranes were measured to evaluate the hydrophilicity of these membranes. The experimental results of water contact angle measurements as a comparative bar chat, and data were included as an index number above each column is depicted in Fig. 5. As expected, the pure PSf (without any additives) exhibited the highest value of contact angle of 87.1°, and addition of additives reduces the contact angle value considerably for the blend membranes with 75.0°, 74.8° and 66.9°, respectively for the 0.50, 0.75 and 1.0 wt% of sPI5. The better affinity between the water droplet and the surface of the membrane results in a smaller contact angle, which enhances surface hydrophilicity. The contact angle values of sPI4 containing membranes were determined to 83.5°, 83.9° and 79.4°, respectively for the 0.5, 0.75 and 1.0 wt% of sPI4. In this case, the reduction was not as large as of sPI5 containing membrane samples. The presence of higher amount of sulfonic acid groups in sPI5 may help in absorption of water molecules, which results in the enhancement of hydrophilicity of the membrane surfaces^10,14,30^.

#### Water uptake

Water uptake values increased considerably with incremental dosages of sPI (Table 2). The water uptake percentage of pure PSf membrane was recorded as 35.2%, and this value increases with the increasing amount of sulfonated additives of sPI4. Similar trend was observed for the blend with sPI5 additives. However, when these two blend membranes were compared; PSf-sPI5 membranes were observed to be higher percentage of water uptake. The enhancement of hydrophilicity, longer and higher pores (Fig. 4) are the main reason of better water retention by the blend membranes. Importantly, the PSf-sPI5 (1 wt%) has the better properties than those of other blended membranes prepared in this study^9,10^.

#### Pure water permeability and anti-fouling study

Hydrophilicity and pore structure are the main governing factors of the permeability study of the UF membranes^9,30^. Time-dependent pure water permeability study was performed using a cross-flow filtration system at 0.4 MPa transmembrane pressure. Initially, each membrane was compacted at 0.5 MPa for 30 min and then the pressure was reduced to 0.4 MPa to obtain the pure water flux. Data was collected at every 15 min interval for 2 h. The variation of pure water permeability of all the membranes is provided in Table 2. The increase in pure water flux in the blend membranes was due to higher hydrophilicity, and better pore structure of the blend membranes. As expected, the pure water permeability of the blend membranes increases with increasing amount of additives upto 1 wt%. Further increase in additives (1.5 wt%), the pure water permeability was found to be decreased; this finding is not very clear at this point. PSf-sPI5 blend membranes showed higher permeability than the PSf-sPI4 membranes. Due to higher sulfonic acid content of sPI5, the blend membranes were having better hydrophilicity and longer and larger pores (Fig. 4), which facilitate higher water retention, facile flow of water through the membranes and eventually gave better permeability^9,30^.

**Figure 6:**
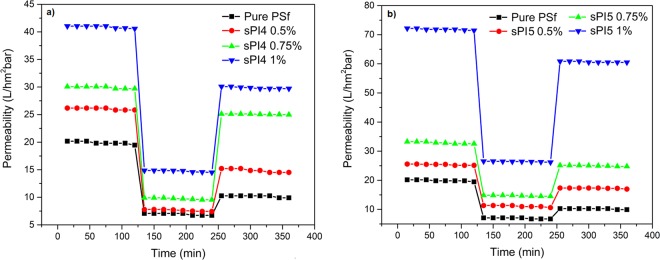
Time dependent pure water permeability, protein BSA permeability, and pure water permeability after washing with water for (**a**) neat membrane (PSf), with increased concentration of sPI4 as sPI4 (0.5 wt%), sPI4 (0.75 wt%), sPI4 (1 wt%), and (**b**) increased concentration of sPI5 as sPI5 (0.5 wt%), sPI5 (0.75 wt%), sPI5 (1 wt%) with an operating pressure of 3 bar at room temperature.

**Figure 7:**
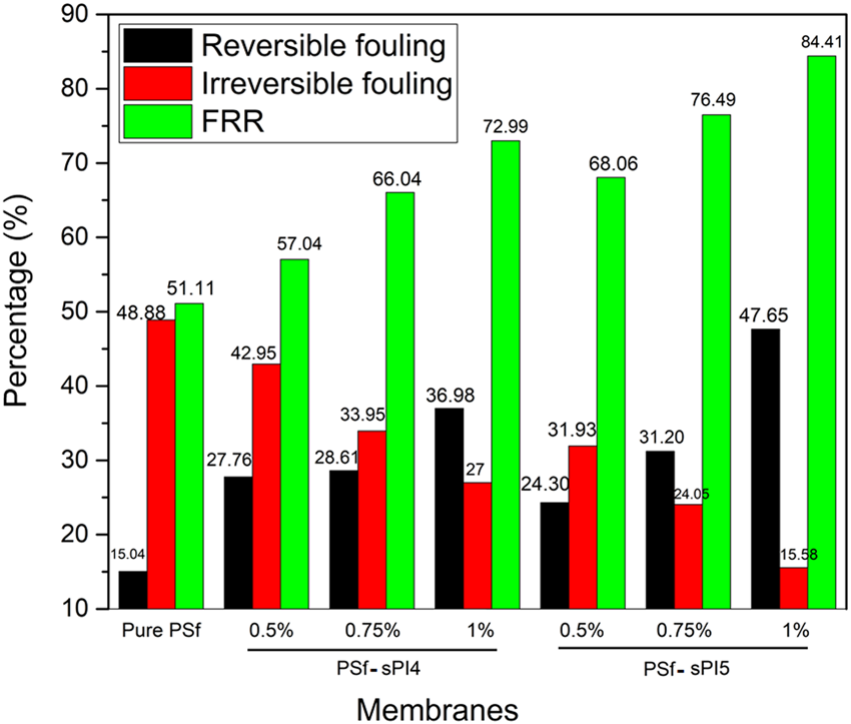
Flux recovery ratio (FRR), reversible fouling and irreversible fouling values for neat membrane (PSf), with increased concentrations (0.5, 0.75 and 1 wt%) of sPI4 or sPI5.

Figure 6(a,b) demonstrated an enhanced BSA permeability study. The blend membranes with an incaresing hydrophilic additives (with the increasing amout of the sulfonated additives), showed an increase in the permeability, which was due to the adsorptive nature of the additives. After cleaning the memebranes, PWF study were performed again. This study suggested there is a decrease in the value of permeability. It could be due to the fact that protein molecules were deposited on the surfaces of membranes, and resulted pore blockage. Usually, proten has a tendency to absorb strongly on less hydrophilic surfaces than the hydrophilic surfaces^9,25,30^. The calculated antifouling parameters namely flux recovery ratio (FRR), reversible fouling (R_r_), and irreversible fouling (R_ir_) are presented in Fig. 7. An increased value of FRR (Fig. 7) suggested better antifouling property for sPI (sPI4 or sPI5) containing membranes than the neat membranes, which could be due to the presence of increasing amount of sulfonated additives in the resulted membranes that also increase the hydrophilicity^10,14,30^ From Fig. 7, it was also found that the R_ir_ value of sPI4 or sPI5 membranes was decreased compare to the neat membranes suggested good filtration life of the blended membranes.

### Surface charge measurement of the membrane

**Figure 8:**
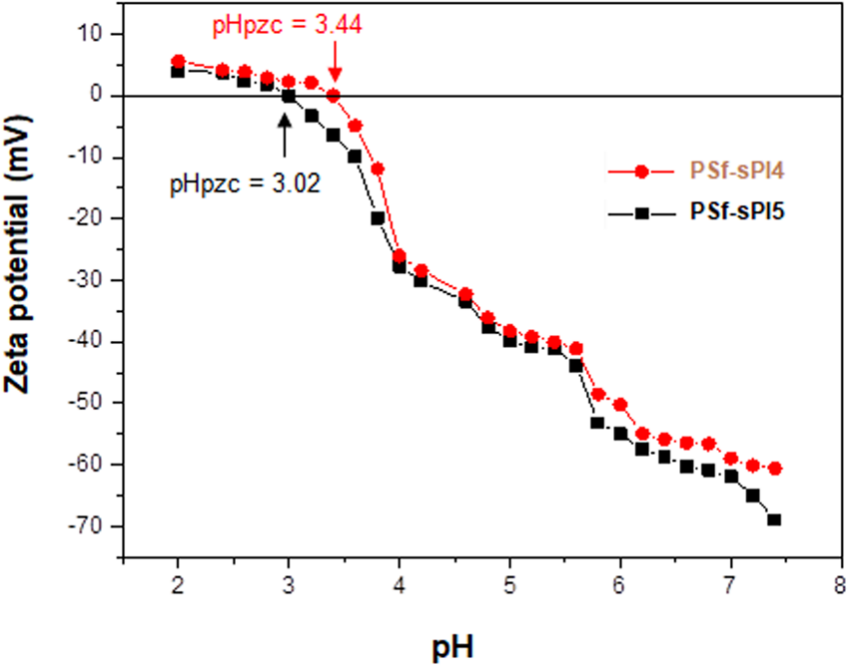
Zeta potential of PSf-sPI4 (1 wt%) and PSf-sPI5 (1 wt%) membranes in different pH.

Zeta potential measurements is an effective technique to study the surface charge of the prepared membranes^27,31^. Figure 8 shows the zeta potential of the two best performing blend membranes PSf-sPI4 (1 wt%) and PSf-sPI5 (1 wt%) in different pH. During the experiment, the pH value of the solution was adjusted in between pH 1.5 to 8 with the addition of 0.1 M HCl and/or 0.1 M KOH. From the Fig. 8, the isoelectric point (IEP) of the PSf-sPI4 (1 wt%) and PSf-sPI5 (1 wt%) blend membranes were calculated to be 3.02 and 3.44, respectively. At this pH, the surface charge becomes zero. The highest zeta potential of −70.7 mV and −60.1 mV was recorded corresponding to pH 7.5 respectively for the PSf-sPI4 (1 wt%) and PSf-sPI5 (1 wt%) membranes, which is much higher than that of pure PSf membrane^27,31^. The surface of blended membranes was negatively charged due to the presence of sulfonic acid groups. As sPI5 has higher DS value (67.6%) than sPI4, the blend membrane with sPI5 (1 wt%) showed higher zeta potential compared to the sPI4 (1 wt%) membrane, which influenced the properties like hydrophilicity, water uptake and eventually affect the performance of heavy metal and protein rejection^10^.

### Heavy metal rejection study

PEUF technique exhibit better efficiency compare to an only UF process. In the PEUF process, water-soluble polydentate ligand (here it was PEI) was used to trap the metal ions to form large size complex^32^. Normally, these complexes are larger than the pores of the membranes and facilitates better removal of metal ions from the polluted water. Among the prepared two sets of blend membranes, PSf-sPI4 (1 wt%) and PSf-sPI5 (1 wt%) were selected for the heavy metal ion rejection studies because of their better hydrophilicity and water permeability.

**Figure 9:**
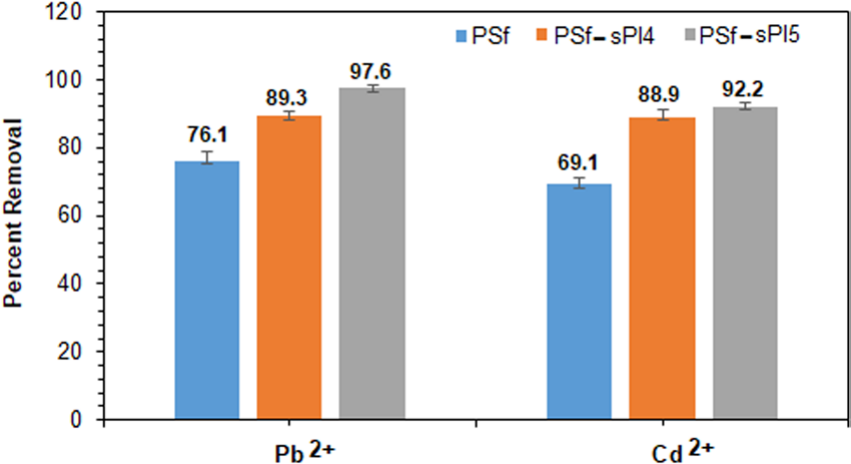
Heavy metal (Pb^2+^ and Cd^2+^) rejection performance of PSf (Neat), PSf-sPI4 (1 wt%) and PSf-sPI5 (1 wt%) blend membranes by PEUF method.

Figure 9 shows the comparative analysis of Pb^2+^ and Cd^2+^ rejection on the pure PSf and blend membranes from the laboratory prepared solution (500 ppm). From Fig. 9, it showed that the blend membranes showed higher removal of Pb^2+^ and Cd^2+^ as compared to the pure PSf membrane. Note that the presence of negatively charged sulfonic acid group on the membrane surface usually facilitates better adsorption of Pb^2+^ and Cd^2+^. In addition, Pb^2+^ has a better affinity towards sulfonic acid group that forms stronger complex with PEI, which attributed for the higher removal of Pb^2+^ in this case^10,12^. During PEUF process the rejection percentage were 89.3% and 97.6% for Pb^2+^ ions, and 88.9% and 92.2% for Cd^2+^ ions respectively for the PSf-sPI4(1 wt%) and PSf-sPI5(1 wt%) membranes. In aqueous solution, the pH is maintained 6.5, so that PEI form chelates with transition metal ions. At lower pH, the metal binds with polybases like PEI, therefore, electron donating imino groups become positively charged due to the protonation and thus unable to form chelates with cations. On the other hand, at higher pH, it forms cadmium and lead hydroxides, which is insoluble in water^33^. A comparison table has been prepared (Table 3) to assess the best performing blend membranes (from this study) with the other reported UF membranes. This will rationalize our efforts and areas of improvement. Mostly non-sulfonated membranes were used for the removal of Pb^2+^ and Cd^2+^ ions. From the Table 3, it is clear that this new blend membrane showed comparable performance and has great potential for improvement (due to the synthetic materials) and utilization in the PEUF/UF technology.

**Table 3:** Comparison of UF membrane separation performance of heavy metals Pb^2+^ and Cd^2+^ with the current work.

Membrane	Heavymetal	Permeability(L h^-1^m^−2^ bar^−1^)	Pressure (bar)	Rejection (%)	Reference
CA/PSf (80/20) + PVP (2 wt%)	Pb^2+^	10 (metal solution)	1	98	^34^
	Pb^2+^	49 (metal solution)	3	98	
	Cd^2+^	15 (metal solution)	1	77	
	Cd^2+^	55 (metal solution)	3	71	
CA/PSf (85/15) + PVP (2.5 wt%)	Cd^2+^	22.5 (metal solution)	3.45	72	^35^
PSf + GO (1 wt%)	Pb^2+^	—	1	98	^36^
	Pb^2+^	52.1 (metal solution)	3	94	
Biochar/PSf membrane	Pb^2+^	33.5 (metal solution)	0.25	> 95.2	^37^
PSf/GO (0.2 wt%)	Pb^2+^	—	4.14	93	^38^
	Cd^2+^	—	4.14	92	
Polysulfone/hydrous ferric oxide (PSf/HFO) membrane (1:1.5)	Pb^2+^	942 (pure water)	0.5	95	^39^
HYP5-F	Cd^2+^	18.4 (pure water)	1	51	^40^
PZM-4 (PSf/NMP= (18/79.2 + 0.72 (ZZSM-5)+ PVP (2 wt%)	Pb^2+^	348.9 (pure water)	4	98	^41^
SA/PSf + PVC (3 wt%)	Cd^2+^		4	95	
	Pb^2+^		1	>90	^42^
	Cd^2+^		1	>90	
PSf-sPI5(1 wt%)	Pb^2+^	72.1 (pure water)	3	97.6	This Work
	Cd^2+^		3	92.2	

### Protein rejection study

**Figure 10:**
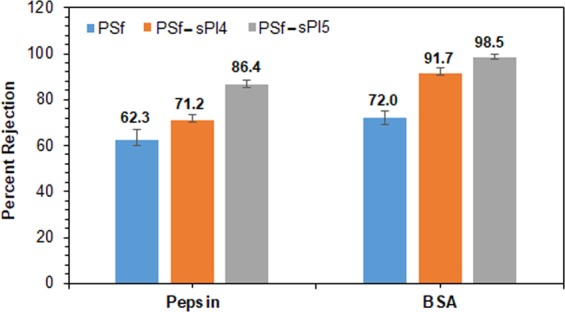
Protein (pepsin and BSA) rejection performance of PSf-sPI4 (1 wt%) and PSf-sPI5 (1 wt%) blend membranes.

Protein removal using membrane techniques is increasingly studied, and receiving higher attention for different prospect due to their potential applications that includes reducing water pollution, purifications of biological enzymes, and recovery of valuable compounds in food industry^43,44^. To study the protein rejection efficacy of these selected membranes, pepsin and BSA solution were prepared with an initial concentration of 1000 ppm. During the study period, the pH of the protein solutions was maintained at 6.8 ± 0.4, as any variation in the pH value can facilitate the fouling nature of the membranes. The comparison bar chat of the protein rejection is presented in Fig. 10. The highest rejection value of 86.4% and 98.5% was calculated for pepsin and BAS, respectively for the blend membranes with 1 wt% sPI5 as additive. The higher rejection of BSA than pepsin was due to the larger size of the BSA molecule, and at the same time, under the filtration condition of pH 6.8 ± 0.4, both pepsin and BSA carry very similar negative charge. Therefore, the two proteins will exhibit repulsive electrostatic interactions between the protein and membranes^45–47^. As PSf-sPI5 (1 wt%) as higher surface charge than that of PSf-sPI4 (1 wt%), the repulsive force might play an affirmative role for better removal of BSA.

## Conclusions

PSf mixed matrix membrane with different composition of sPI4 and sPI5 additives were prepared using phase inversion method. The membranes showed well-formed asymmetric structure with dense top layer and porous layer. The addition of organic additives has resulted interconnected finger like porous structure and enhanced hydrophilic surface. The addition of PVP has resulted in micro porous within the membrane structure with increased permeability. The contact angle of the membrane decreased with increasing sulfonated additive (sPI4 or sPI5), indicating increased hydrophilicity of the new membranes. The effects of organic additives on the flat sheet structure and performance were analyzed and reported. The blended flat sheet membranes showed an increase in pure water flux, porosity, and better hydrophilicity, hence better water content. Filtration experiments were conducted to assess the applicability and performance of the membrane for heavy metal (Pb^2+^ and Cd^2+^) and protein rejection (Pepsin and BSA). The rejection studies indicated improvement in heavy metal and protein rejections with increasing the concentration of sulfonated additives. More study will be required to optimize the membrane fabrication process and the studied membranes particularly PSf-sPI5 (1 wt%) which has great potential in different filtration technologies.

## Supplementary information

Supplementary information.
