# Supplementary material for: Assessment of sulfonated homo and co-polyimides incorporated polysulfone ultrafiltration blend membranes for effective removal of heavy metals and proteins
Source: Sci Rep. 2020 Apr 27;10:7049. doi: 10.1038/s41598-020-63736-8 (PMC7184734; doi:10.1038/s41598-020-63736-8)
Supplement: Supplementary file 1 — Supplementary information. [file 41598_2020_63736_MOESM1_ESM.docx]

**Assessment of sulfonated homo and co-polyimides incorporated polysulfone ultrafiltration blend membranes for effective removal of heavy metals and proteins**

Mohammad A. Jafar Mazumder*^1^, Panchami H. Raja^2^, Arun M. Isloor^2^, Muhammad Usman^3^, Shakhawat H. Chowdhury^4^, Shaikh A. Ali^1^, Inamuddin^5,6^, Amir Al-Ahmed^7^

^1^Chemistry Department, King Fahd University of Petroleum & Minerals, Dhahran 31261, Saudi Arabia

^2^Membrane Technology Laboratory, Chemistry Department, National Institute of Technology Karnataka, Surathkal, Mangalore 575 025, India

^3^Center for Research Excellence in Nanotechnology (CENT), King Fahd University of Petroleum & Minerals, Dhahran 31261, Saudi Arabia.

^4^Department of Civil and Environmental Engineering, King Fahd University of Petroleum & Minerals, Dhahran 31261, Saudi Arabia

^5^Chemistry Department, Faculty of Science, King Abdulaziz University, Jeddah 21589, Saudi Arabia

^6^Advanced Functional Materials Laboratory, Department of Applied Chemistry, Faculty of Engineering and Technology, Aligarh Muslim University, Aligarh- 202 002, India

^7^Center of Research Excellence in Renewable Energy, King Fahd University of Petroleum & Minerals, Dhahran 31261, Saudi Arabia.


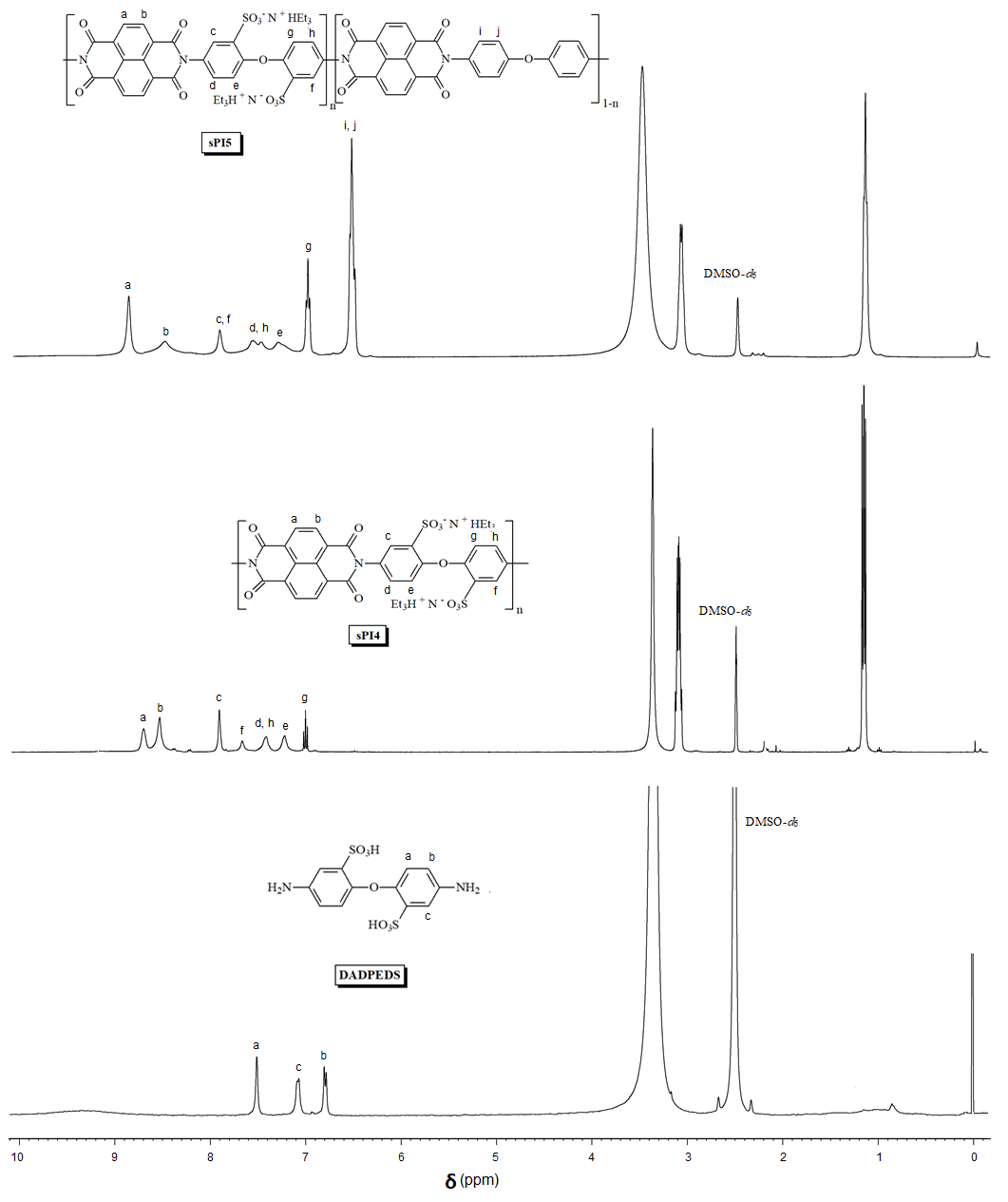


**Fig. S1:** ^1^H NMR spectra of a) DADPEDS, b) sPI4, and c) sPI5.

**^1^H NMR Data:**

**DADPEDS:** δ_H_ (DMSO-*d*_6_; Few drops of Et_3_N was added to dissolve): 6.80 (1H, d), 7.08 (1H, d), 7.51 (1H, s), (residual H in DMSO-*d*_6_ at 2.50 ppm).

**sPI4:** δ_H_ (DMSO-*d*_6_): 7.01(1H), 7.23 (1H), 7.42 (1H), 7.54 (1H), 7.66 (1H), 7.91 (1H), 8.58 (2H), 8.74 (2H) (residual H in DMSO-*d*_6_ at 2.50 ppm);

**sPI5:** δ_H_ (DMSO-*d*_6_): 6.52(2H), 6.56 (2H), 6.99 (2H), 7.32 (2H), 7.50 (2H), 7.59 (2H), 7.94 (2H), 8.74 (4H), 8.98 (4H) (residual H in DMSO-*d*_6_ at 2.50 ppm).


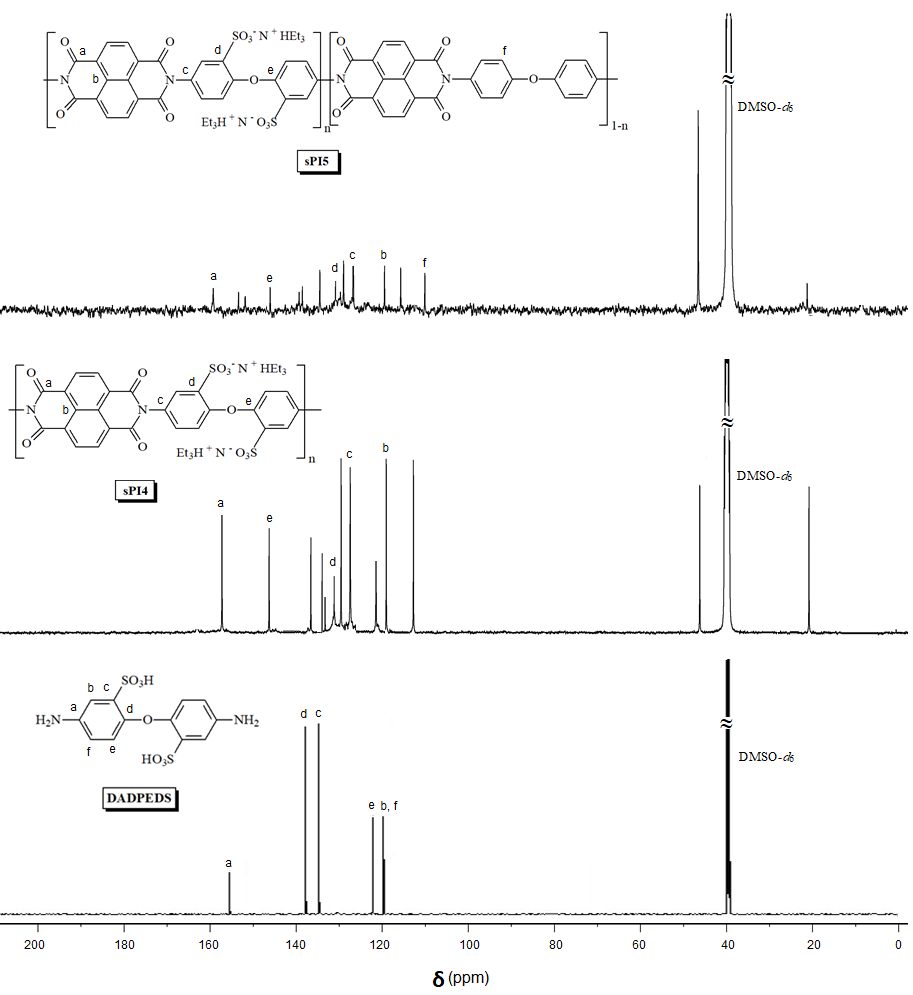


**Fig. S2:** ^13^C NMR spectra of a) DADPEDS, b) sPI4, and c) sPI5.

**^13^C NMR Data:**

**DADPEDS:** δ_C_ (DMSO-*d*_6_): 119.9 (2C), 120.8 (2C), 122.7 (2C), 135.6 (2C), 138.4 (2C), 155.5 (2C).

**sPI4:** δ_C_ (DMSO-*d*_6_): 21.1 (1C), 45.7 (1C), 117.8 (2C), 120.1 (2C), 121.6 (1C), 126.3 (2C), 130.5 (2C), 132.9 (2C), 134.0 (1C), 135.2 (4C), 138.3 (4C), 146.8 (2C), 158.2 (4C).

**sPI5:** δ_C_ (DMSO-*d*_6_): 20.9 (1C), 44.8 (1C), 115.6 (2C), 118.4 (4C), 120.5 (6C), 127.2 (5C), 128.7 (2C), 131.4 (3C), 133.8 (2C), 135.2 (8C), 138.3 (4C), 139.6 (8C), 147.1 (2C), 152.4 (2C), 158.6 (6C), 159.6 (2C).


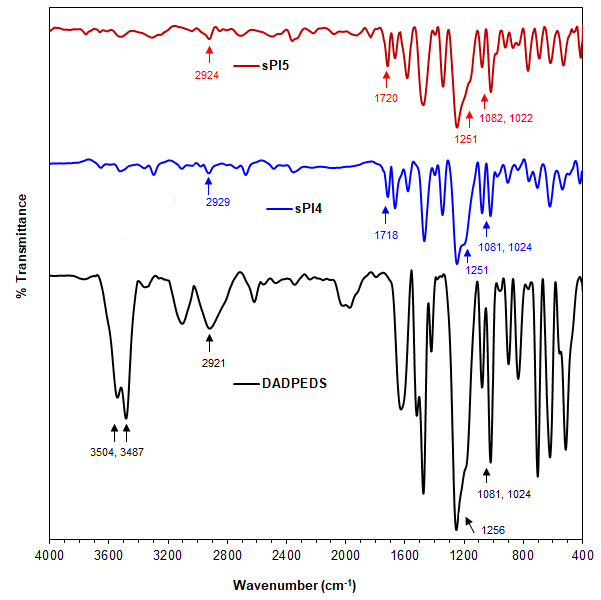


**Fig. S3:** FT-IR spectra of a) DADPEDS, b) sPI4, and c) sPI5.

**FT-IR Data:**

**DADPEDS:** ν_max._ (neat) 3504, 3487 (NH_2_ stretch), 3087, 2921 (aromatic, CH stretch), 1629, 1523, 1478, 1424, 1256 (SO_3_H stretch), 1081, 1024 (C-O-C stretch), 903, 837, 705 and 623 cm^-1^.

**sPI4:** ν_max._ (neat) 2929 (aromatic, CH stretch), 1718 (C=O stretch), 1669, 1582, 1472, 1349, 1251 (asymmetric SO_3_^-^ stretch), 1081, 1024 (C-O-C stretch), 908, 839, 766, 701 and 624 cm^-1^.

**sPI5:** ν_max._ (neat) 2924 (aromatic, CH stretch), 1720 (C=O stretch), 1670, 1587, 1479, 1345, 1251 (asymmetric SO_3_^-^ stretch), 1082, 1022 (C-O-C stretch), 925, 873, 771, 695 and 619 cm^-1^.
